# Comparison of a hepatitis C core antigen assay to nucleic acid amplification testing for detection of hepatitis C viremia in a US population

**DOI:** 10.1128/spectrum.00975-24

**Published:** 2024-10-09

**Authors:** Ian L. Gunsolus, John Prostko, Sandra Pearce, Biniam Degaga, Scott Eickstead, Russ Taylor, Jessica Grieshaber, Kyle Richard, Anne Hoffman, Aneta Pekalska, David Daghfal

**Affiliations:** 1 Department of Laboratory Medicine and Pathology, HealthPartners, Minneapolis, Minnesota, USA; 2 Core Diagnostics, Abbott Laboratories, Abbott Park, Illinois, USA; Indian Institute of Science, Bangalore, Karnataka, India

**Keywords:** hepatitis C virus, core antigen, nucleic acid amplification testing

## Abstract

The prevalence of hepatitis C virus (HCV) infection in the United States has increased over the past decade despite the development of effective direct-acting antiviral treatments. To meet the World Health Organization’s (WHO) goal of eliminating HCV infection by 2030, transmission events must be reduced. Currently, infection screening relies on detection of HCV antibodies, with nucleic acid amplification testing (NAAT) used to confirm HCV viremia and monitor changes in viral load. However, the seroconversion window for detection of HCV antibodies is long, averaging 6 weeks, with delayed seroconversion common in co-infected and immunosuppressed populations. Testing for HCV core antigen, which is present approximately 5 weeks before HCV antibodies, holds promise for earlier detection of HCV infection. It may also hold promise as a cheaper, more accessible, and more rapid alternative to NAAT for infection confirmation. Here, we evaluated the agreement between a research-use HCV Core Antigen Assay and NAAT among US patients receiving clinically indicated NAAT. Among 412 specimens, the overall concordance was 97.1%, with a positive percent agreement of 95.5%. Discrepancies primarily occurred among patients with chronic HCV and low viral loads; 11/12 discrepancies showed viral loads <4,000 IU/mL. Among patients being screened for HCV infection (i.e., excluding those undergoing NAAT for serial monitoring of a previously diagnosed infection), the positive percent agreement was 97.0%. Among patients undergoing serial testing, changes in HCV Core Antigen Assay signal-to-cut-off values were generally correlated with changes in the viral load. Results suggest that the research-use HCV Core Antigen Assay studied here may reliably detect and/or confirm HCV infection.

**IMPORTANCE:**

A research-use HCV Core Antigen Assay showed high concordance with nucleic acid amplification testing for the detection of current hepatitis C infection. The assay may enable more rapid and lower-cost detection and/or confirmation of hepatitis C infection.

## INTRODUCTION

The target of eliminating hepatitis C virus (HCV) infection by 2030 was established by the World Health Organization in 2016 and adopted by many member countries’ specific health organizations (1). Major strides have been made in curtailing infections and treating those infected. While the prevalence of HCV in the United States remains 1–2%, rates have shown a rising trend since 2017 (2), exacerbated by the COVID-19 pandemic and associated with a decreasing rate of direct-acting antiviral treatment (3). HCV antibody immunoassays are the first line of screening for HCV, and US guidelines currently require confirmation of infection via nucleic acid amplification testing (NAAT) (4). The seroconversion window for detection of antibodies to HCV has been reported to average 6 weeks (5), with delayed seroconversion common in co-infected and immunocompromised groups (6). With the Centers for Disease Control and Prevention reporting that the number of acute HCV cases has doubled since 2014 in the United States (7), closing the seroconversion detection window should become a public health priority. Turnaround time for NAAT is often multiple days, potentially increasing rates of patient loss to follow-up and causing delays in counseling and treatment. More methods to detect and/or confirm both chronic and acute HCV viremia rapidly and in a cost-effective manner are needed. Testing for HCV core antigen, which is typically detected approximately 5 weeks before antibodies (8), could potentially help detect HCV viremia earlier and streamline clinical follow-up. The use of HCV core antigen testing would be in line with the recent calls from the National Institutes of Health for the elimination of the approximately 15,000 annual deaths from this disease still occurring in the United States in 2023 and enabling quicker linkage to treatment protocols. Depending on its clinical performance, HCV core antigen testing could also represent a more accessible and potentially cheaper alternative to NAAT to confirm acute HCV infection. The key objectives of this study were to evaluate the sensitivity and concordance to NAAT of a research-use-only HCV Core Antigen Assay in a US-based population.

## MATERIALS AND METHODS

We assessed concordance between the research use-only HCV Core Antigen Assay (Abbott Diagnostics) and the Food and Drug Administration (FDA)-cleared Aptima HCV Quant Dx Assay for HCV RNA quantitation (Hologic).

HCV Core Antigen Assay testing was performed on the Alinity i instrument (Abbott Diagnostics) using residual specimens submitted to our laboratory (Minneapolis, MN, USA) for HCV viral load quantitation. Testing was performed on the same day as viral load quantitation. All specimens were also screened for HCV antibodies using the ARCHITECT or Alinity anti-HCV assay (Abbott Diagnostics) either in the course of clinically indicated testing using fresh specimens or for study purposes using residual specimens.

The HCV Core Antigen Assay is a qualitative chemiluminescent immunoassay with values ≥1 S/CO considered reactive and <1 S/CO non-reactive.

The Aptima HCV Quant Dx Assay is a quantitative transcription-mediated amplificated (TMA) assay performed on the Panther System with a limit of quantitation of 10 IU/mL.

Residual specimens were obtained via random sampling of specimens submitted to our laboratory for HCV RNA quantitation, regardless of the clinical indication. Specimens were categorized as either *monitoring* or *screening* according to the HCV RNA quantitation testing history; those with a history of HCV RNA quantitation testing within our healthcare system were presumed to be ordered for monitoring a known infection, while those without such testing history were presumed to be ordered to screen for infection. Importantly, some screening tests may actually represent monitoring of a known HCV infection if testing was performed outside our healthcare system and not available in the electronic medical record.

For a subset of specimens, we attempted to amplify and sequence the 5′ untranslated region of 750 base pairs coding for the HCV Core protein. RNA was extracted from available plasma using the QIAamp DNA Blood Mini Kit (QIAGEN). Samples were eluted in 50 µL elution buffer and 15 µL was used for reverse transcription polymerase chain reaction (RT-PCR). RT-PCR was performed using Invitrogen SuperScript IV One-Step RT-PCR reagents (Invitrogen) and gene-specific primers in 50 µL total volume at 45 cycles.

We also assessed the analytical performance of the HCV Core Antigen Assay through precision and dilution recovery studies. Precision was assessed by repeated testing of negative human plasma control and two positive controls prepared with recombinant HCV core antigen in a buffer; 4–10 replicates were tested per day for 3 days. Dilution recovery was evaluated by preparing four dilutions of a residual specimen with detectable HCV viral load.

## RESULTS

Specimens were collected between March 2022 and November 2023. A total of 452 specimens were included in the concordance study; all, but one, showed detectable HCV antibodies, suggesting prior exposure to HCV. The lone specimen with non-detectable HCV antibodies was obtained from a patient with chronic HCV infection who previously (6 years prior) showed detectable HCV antibodies and, at the time of this study, had HCV viral load >10,000,000 IU/mL.

The mean (standard deviation) antibody signal-to-cut-off value was 10.6 (5.5), with values ≥ 1 considered reactive for HCV antibodies and values <1 considered non-reactive.

**Fig 1:**
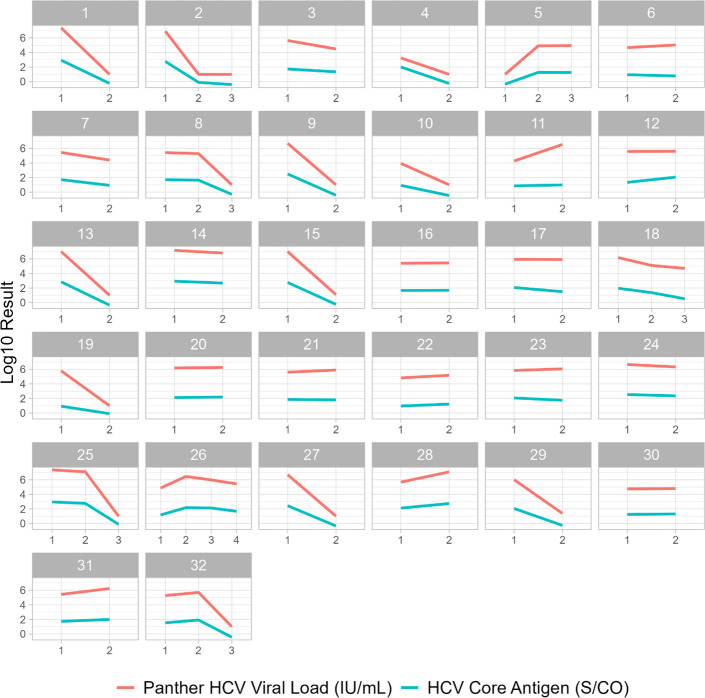
Serial HCV viral load and HCV Core Antigen Assay signal-to-cut-off values among 32 patients contributing more than one specimen. The *x*-axis denotes serial specimen counts; a value of 1 denotes the first specimen collected; and subsequent values are ordered chronologically by collection date. The *x*-axis scale is arbitrary; elapsed time between serial collections ranged from 6 to 546 days. The *y*-axis denotes the log_10_ value of the HCV viral load (top line, orange) or HCV Core Antigen Assay signal-to-cut-off value (bottom line, blue).

Among the 452 specimens studied, 412 were obtained from unique patients. A total of 32 patients contributed more than one specimen to the cohort due to multiple instances of clinically indicated HCV RNA quantitation during the study period (25 patients contributed two specimens; six contributed three specimens; and one contributed four). Serial results obtained from non-unique patients are shown in Fig. 1. These showed similar magnitude changes in both HCV viral load and core antigen signal-to-cutoff values in a majority of patients. Among unique patients, the mean (standard deviation) age was 50.0 (16.7) years with a median of 51 years and a range of 3–87 years. The fraction of females to males was 45/55%. The fraction of *monitoring* and *screening* specimens, as defined in the Methods section, was 44/56%.

The concordance between HCV viral load and core antigen testing among specimens contributed by unique patients is summarized in Table 1. The overall percent agreement was 97.1% (400/412), with a positive percent agreement of 95.5% (252/264) and a negative percent agreement of 100% (148/148). Cohen’s kappa [95% confidence interval (CI)] was 0.938 (0.903–0.972). Considering HCV viral load testing to be the gold standard method to identify current HCV infection, the sensitivity (95% CI) of the HCV Core Antigen Assay was 95.5% (92.0–97.5%) across the full cohort (i.e., among both *screening* and *monitoring* specimens). Specificity (95% CI) was 100.0% (96.8–100.0%). Among *screening* tests (i.e., those ordered for patients without a history of HCV viral load testing), the overall percent agreement was 98.3% (228/232), with sensitivity (95% CI) of 97.0% (92.1–99.0%); see Table 2. Among *monitoring* tests (i.e., those ordered for patients with a history of HCV viral load testing), the overall percent agreement was 95.6% (172/180), with sensitivity (95% CI) of 93.8% (87.8–97.1%); see Table 3.

**TABLE 1:** Concordance between HCV viral load and HCV Core Antigen Assay among all residual specimens submitted for HCV viral load testing from unique patients

		HCV viral load		
		Positive	Negative	Total
HCV core antigen	Positive	252	0	252
	Negative	12	148	160
	Total	264	148	412

**TABLE 2:** Concordance between HCV viral load and HCV Core Antigen Assay among residual specimens submitted for HCV viral load testing among unique patients without HCV viral load testing history (HCV infection screening)

		HCV viral load		
		Positive	Negative	Total
HCV core antigen	Positive	131	0	131
	Negative	4	97	101
	Total	135	97	232

**TABLE 3:** Concordance between HCV viral load and HCV Core Antigen Assay among residual specimens submitted for HCV viral load testing among unique patients with HCV viral load testing history (HCV infection monitoring)

		HCV viral load		
		Positive	Negative	Total
HCV core antigen	Positive	121	0	121
	Negative	8	51	59
	Total	129	51	180

Discordant HCV viral load and core antigen results were observed for 12 unique patients. All discordant results showed detectable HCV viral load and non-reactive HCV core antigen results (Table 4). The range of viral loads was 12 to 18,164 IU/mL, with a median of 382 IU/mL. Significantly, 2/12 discordant results were obtained from patients who showed subsequent HCV viral load <10 IU/mL within approximately 1 month; a third discordant result showed HCV viral load <10 IU/mL within 3.5 months. Viral sequencing to obtain the HCV genotype was attempted for five specimens but was unsuccessful due to insufficient viral load; the remaining seven specimens had insufficient volume to attempt sequencing.

**TABLE 4:** Characteristics of discordant HCV viral load and HCV Core Antigen Assay results among unique patients^*a*^

HCV viral load (IU/mL)	HCV core antigen (S/CO)
12	0.43
12^*b*^	0.63
17	0.38
43^*b*^	0.37
53	0.57
236	0.56
527	0.56
1182^*c*^	0.39
1917	0.72
2073	0.86
3893	0.92
18164^*d*^	0.75

^*a*^ Viral load limit of detection: 10 IU/mL. HCV Core Antigen Assay: ≥1 S/CO reactive, <1 S/CO non-reactive.

^*b*^ Specimens showed HCV viral loads < 10 IU/mL within 1 month.

^*c*^ Specimen showed HCV viral load < 10 IU/mL within 3.5 months.

^*d*^ Attempted viral sequencing via RT-PCR, which was unsuccessful.

**Fig 2:**
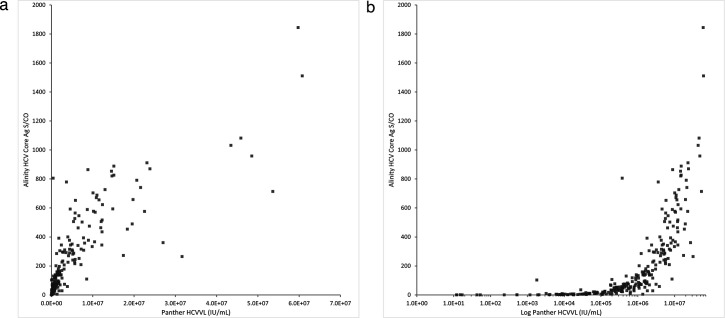
(**a**) Correlation of HCV Core Antigen Assay S/CO vs HCV viral load among unique patients with detectable HCV viral loads (*n* = 264). Pearson’s *r* = 0.831. (**b**) Linear-log transformation of HCV Core Antigen Assay S/CO vs HCV viral load among unique patients with detectable HCV viral loads (*n* = 264).

The correlation between the HCV Core Antigen Assay S/CO value and HCV viral load values among specimens with detectable viral load is shown in Fig. 2a; a linear-log transformation is shown in Fig. 2b. Pearson’s correlation coefficient was 0.831.

The precision of the HCV Core Antigen Assay was assessed using control materials (Table 5). The coefficient of variation ranged between 7 and 21% depending on the sample S/CO level tested. Dilution recovery averaged 98.2% (range 90.2–107.3%, data not shown).

**TABLE 5:** Precision of the HCV Core Antigen Assay using control materials

	Control 1	Control 2	Control 3
Mean/S/CO	0.32	1.47	20.25
SD/S/CO	0.07	0.15	1.41
CV%	21.34	10.24	6.96

## DISCUSSION

Substantial agreement was observed between the research use-only HCV Core Antigen Assay and an FDA-cleared HCV viral load assay, consistent with previous reports (9), including in two pediatric cases (ages 3 and 18). This suggests that the HCV Core Antigen Assay may be appropriate to confirm HCV infection in conjunction with HCV antibody testing. False-negative HCV core antigen results were primarily observed in cases of treated chronic HCV infection with low viral loads. Results suggest a limited risk of falsely ruling out HCV infection among patients with viral loads typically observed during diagnostic testing for acute and chronic untreated HCV infection. No false-positive results were observed, suggesting a limited risk of initiating unnecessary treatment based on erroneous HCV core antigen results. Correlation between HCV Core Antigen Assay signal-to-cutoff values and viral load values across the study population, as well as relative changes observed among serial tests obtained from a subset of the population, suggest that HCV Core Antigen Assay signal is generally proportional to the viral load. Some exceptions were observed; these primarily showed lower core antigen signal than expected for the viral load.

NAAT is currently recommended to confirm acute HCV infection (10) but is not available in many clinical laboratories owing to limited resources and/or technical knowledge necessary to perform molecular diagnostic testing. Consequently, healthcare systems commonly use reference laboratory services to perform this testing, resulting in a multi-day delay to confirm acute infection following a positive HCV antibody screening result. The availability of an HCV core antigen immunoassay compatible with instrumentation also used to perform HCV antibody screening would significantly reduce the time between initial screening and confirmatory testing. Potential benefits of rapid, local testing for HCV core antigen include reduced patient anxiety following presumptively positive antibody screening results, reduced time to specialist referral (11), more rapid initiation of treatment (11), and reduction in potential transmission events. To the extent that the adoption of HCV core antigen testing could reduce the need for secondary specimen collection prior to confirmatory testing (i.e., facilitate single-step as opposed to two-step testing), cost-effectiveness is expected to increase (12, 13). NAAT may or may not be performed to confirm HCV infection in cases of discrepant antibody and core antigen results according to assay sensitivity and local infection prevalence. NAAT would remain necessary to establish baseline viral load values or monitor serial changes in the viral load, though HCV treatment strategies employing minimal treatment monitoring have proven to achieve similar rates of sustained virologic response as those employing more intensive monitoring (14).

The HCV Core Antigen Assay could be incorporated into the HCV diagnostic testing cascade in at least two ways. First, HCV core antigen testing could, if positive, confirm acute HCV infection in patients with positive HCV antibody screening results, thereby reducing the time to infection confirmation, as noted above; if negative, NAAT may or may not be necessary, according to the complete clinical picture, to discriminate between the absence of infection and false-negative HCV core antigen results. Following confirmation of HCV infection through core antigen testing, NAAT would be required for viral load assessments, where turnaround time is of less clinical significance compared to initial diagnostic testing. Alternatively, the HCV Core Antigen Assay could be used in parallel with HCV antibody testing for initial screening, increasing sensitivity for early infection, prior to seroconversion (15). In the latter scenario, observation of parallel, positive HCV antibody and antigen results would confirm acute HCV infection. Discrepant antibody and core antigen results would suggest either acute infection prior to seroconversion (antibody negative, core antigen positive) or resolving infection with non-detectable or very low viral load (antibody positive, core antigen negative). This parallel testing scheme would increase screening costs owing to the use of two screening tests in place of one employed today; the additional cost of the HCV Core Antigen Assay must be weighed against the clinical and economic values of enhanced HCV infection detection, particularly in the acute phase prior to seroconversion, to evaluate the feasibility of this screening scheme.

Limitations of our study include a retrospective design and limited clinical data collection, including patient demographics and indication for HCV viral load testing. We are also unable to comment on the performance of the HCV Core Antigen Assay across HCV genotypes, as genotyping information could not be obtained. Finally, we are unable to analyze the cost-effectiveness of the HCV Core Antigen Assay due to its research use-only (non-commercial) status and further study is required to identify the optimal scheme for incorporating the assay into HCV screening paradigms.

Overall, our results show a research use-only HCV Core Antigen Assay with >95% agreement to NAAT among patients with HCV viremia, suggesting that the former may reliably detect and/or confirm HCV infection.

## References

[1] Fleurence RL, Collins FS. 2023. A national hepatitis C elimination program in the United States: a historic opportunity. JAMA 329:1251–1252. doi:10.1001/jama.2023.3692 36892976

[2] Numbers and rates of reported cases of acute hepatitis C virus infection, by demographic characteristics — United States, 2017–2021. 2021. Centers for Disease Control and Prevention

[3] Nguyen VH, Kam L, Yeo YH, Huang DQ, Henry L, Cheung R, Nguyen MH. 2022. Characteristics and treatment rate of patients with hepatitis C virus infection in the direct-acting antiviral era and during the COVID-19 pandemic in the United States. JAMA Netw Open 5:e2245424. doi:10.1001/jamanetworkopen.2022.45424 36477481 PMC9856330

[4] Schillie S, Wester C, Osborne M, Wesolowski L, Ryerson AB. 2020. CDC recommendations for hepatitis C screening among adults — United States, 2020. MMWR Recomm Rep 69:1–17. doi:10.15585/mmwr.rr6902a1 PMC7147910 32271723

[5] Netski DM, Mosbruger T, Depla E, Maertens G, Ray SC, Hamilton RG, Roundtree S, Thomas DL, McKeating J, Cox A. 2005. Humoral immune response in acute hepatitis C virus infection. Clin Infect Dis 41:667–675. doi:10.1086/432478 16080089

[6] Thomson EC, Nastouli E, Main J, Karayiannis P, Eliahoo J, Muir D, McClure MO. 2009. Delayed anti-HCV antibody response in HIV-positive men acutely infected with HCV. AIDS 23:89–93. doi:10.1097/QAD.0b013e32831940a3 19050390 PMC2646374

[7] Number of reported cases of acute hepatitis C virus infection and estimated infections — United States, 2014–2021. 2021. Centers for Disease Control and Prevention

[8] Wang Y, Jie W, Ling J, Yuanshuai H. 2021. HCV core antigen plays an important role in the fight against HCV as an alternative to HCV-RNA detection. J Clin Lab Anal 35:e23755. doi:10.1002/jcla.23755 33788295 PMC8183919

[9] Kyuregyan KK, Malinnikova EY, Soboleva NV, Isaeva OV, Karlsen AA, Kichatova VS, Potemkin IA, Schibrik EV, Gadjieva OA, Bashiryan BA, Lebedeva NN, Serkov IL, Yankina A, Galli C, Mikhailov MI. 2019. Community screening for hepatitis C virus infection in a low-prevalence population. BMC Public Health 19:1038. doi:10.1186/s12889-019-7388-7 31375104 PMC6679455

[10] Bhattacharya D, Aronsohn A, Price J, Lo Re V, Panel A-I. 2023. Hepatitis C guidance 2023 update: AASLD-IDSA recommendations for testing, managing, and treating hepatitis C virus infection. Clin Infect Dis:ciad319. doi:10.1093/cid/ciad319 37229695

[11] Torrecillas M, Gómez-Muñoz N, Ocete MD, Cuevas PR, Madrid MD, González EO, Cardona CG, García-Deltoro M. 2022. One-step diagnosis strategy together with multidisciplinary telematics referral perform an effective approach for identifying and treating patients with active hepatitis C infection. Ann Hepatol 27:100542. doi:10.1016/j.aohep.2021.100542 34571265

[12] Marcellusi A, Mennini FS, Ruf M, Galli C, Aghemo A, Brunetto MR, Babudieri S, Craxi A, Andreoni M, Kondili LA. 2022. Optimizing diagnostic algorithms to advance hepatitis C elimination in Italy: a cost effectiveness evaluation. Liver Int 42:26–37. doi:10.1111/liv.15070 34582627 PMC9292516

[13] Jülicher P, Chulanov VP, Pimenov NN, Chirkova E, Yankina A, Galli C. 2019. Streamlining the screening cascade for active hepatitis C in Russia: a cost-effectiveness analysis. PLoS One 14:e0219687. doi:10.1371/journal.pone.0219687 31310636 PMC6634401

[14] Solomon SS, Wagner-Cardoso S, Smeaton L, Sowah LA, Wimbish C, Robbins G, Brates I, Scello C, Son A, Avihingsanon A, Linas B, Anthony D, Nunes EP, Kliemann DA, Supparatpinyo K, Kityo C, Tebas P, Bennet JA, Santana-Bagur J, Benson CA, Van Schalkwyk M, Cheinquer N, Naggie S, Wyles D, Sulkowski M. 2022. A minimal monitoring approach for the treatment of hepatitis C virus infection (ACTG A5360 [MINMON]): a phase 4, open-label, single-arm trial. Lancet Gastroenterol Hepatol 7:307–317. doi:10.1016/S2468-1253(21)00397-6 35026142 PMC8920770

[15] Couroucé AM, Le Marrec N, Bouchardeau F, Razer A, Maniez M, Laperche S, Simon N. 2000. Efficacy of HCV core antigen detection during the preseroconversion period. Transfusion 40:1198–1202. doi:10.1046/j.1537-2995.2000.40101198.x 11061855

